# The Value of Sacral Reflex and Sympathetic Skin Reflex in the Diagnosis of Multiple System Atrophy P-Type

**DOI:** 10.1155/2021/6646259

**Published:** 2021-01-20

**Authors:** Xiaohang Li, Chengju Wang, Xueming Zhang, Wanli Zhang, Binbin Deng, Xun Wang, Huanjie Huang

**Affiliations:** ^1^Yanzhou Branch of Affiliated Hospital of Jining Medical University, Jining 272100, China; ^2^Department of Neurology, The First Affiliated Hospital of Wenzhou Medical University, Wenzhou 325000, China; ^3^The First People's Hospital of Ningyang County, Taian 271000, China

## Abstract

**Objectives:**

To observe the characteristics of sacral reflex and sympathetic skin reflex in patients with Parkinson's disease (PD) and multiple system atrophy P-type (MSA-P) and to analyze their value as a differential diagnostic method.

**Methods:**

The data of 30 healthy people, 58 PD patients, and 52 MSA-P patients from the First Affiliated Hospital of Wenzhou Medical University were collected. Electrophysiological bulbocavernosus reflex (BCR) and sympathetic skin response (SSR) were evaluated using the Keypoint EMG/EP system. The latency period, amplitude, and extraction rate of BCR and SSR were compared between the control, PD, and MSA-P groups.

**Results:**

The incidence of the related autonomic damage in the PD group was lower than that of the MSA-P group. For BCR, the latency period was shorter and the amplitude and elicitation rates were lower in the PD group than in the MSA-P group. For SSR, the latency period was longer in the MSA-P and PD groups than in the control group, but the difference was not statistically significant.

**Conclusion:**

SSR cannot be used to assess autonomic nerve function. PD patients can have clinical symptoms similar to those of MSA-P patients, but the incidence is lower. Both MSA-P and PD patients have a damage to the BCR arc, but the MSA-P patients have a more severe damage.

## 1. Introduction

The early differential diagnosis of multiple system atrophy P (MSA-P) and Parkinson's disease (PD) has always been challenging. In the past, it was believed that sacral reflex dysfunction was the main basis for distinguishing PD from MSA-P [1–4]. However, as sacral reflex dysfunction in PD patients is gradually recognized, the distinction between MSA-P and PD has become more difficult. Therefore, the objective evaluation of sacral reflex dysfunction has attracted the attention of clinicians again. Although both MSA-P and PD have symptoms of sacral nerve involvement, due to the different anatomical parts involved, the severity of the involvement differs. Some electrophysiological examinations can make a certain distinction between the two conditions [5–7].

Sympathetic skin response (SSR) is an objective examination method used to assess the sympathetic nervous system. It mainly reflects the functional status of the sympathetic nerve fibers after the ganglion. Damage to any link in the conduction pathway can cause abnormalities in SSR, which can reflect the functional status of the sympathetic nerves more comprehensively.

The bulbocavernosus reflex (BCR) is closely related to the rectal, bladder, and sexual functions. Our previous research results have showed that the amplitude of BCR in MSA-P patients was decreased and the latency period prolonged. Therefore, we aimed to observe the characteristics of BCR and SSR in PD and MSA-P patients and to analyze their value as a differential diagnostic method.

## 2. Materials and Methods

### 2.1. Subjects

The data of 110 outpatients and inpatients of the Department of Neurology of the First Affiliated Hospital of Wenzhou Medical University from May 2013 to September 2018 were collected. Among them, there were 58 patients with PD and 52 patients with MSA-P. The PD patients enrolled in this study were those who met the diagnostic criteria established by the Movement Disorders and Parkinson's Disease Group of the Neurology Branch of the Chinese Medical Association [8]. The MSA-P patients were those who met the 2008 Gilman Diagnostic criteria [9]. Thirty healthy subjects were also recruited from the Physical Examination Center of the First Affiliated Hospital of Wenzhou Medical University. This study was approved by the ethics committee of the First Affiliated Hospital of Wenzhou Medical College, and all subjects provided written informed consent prior to study participation.

### 2.2. Measurement Methods

#### 2.2.1. Operation Method of BCR

The BCR test uses the Keypoint EMG/evoked potential meter produced by Vidi, Denmark. During the inspection, the room should be kept quiet with a temperature of approximately 24°C. The subject was required to cooperate with the examination in a relaxed state, and the subject's skin temperature should remain >32°C. The subject took the stone cutting position and placed the ground wire on the ankle. In female subjects, measurement was performed by placing the stimulating cathode on the clitoris and the anode was placed on the labia. In male subjects, the stimulating electrode used was a ring electrode placed on the penile body. The recording electrode was a concentric needle electrode, and the electrodes were inserted into the left and right cavernous muscles in sequence. The stimulation was started with a square wave of 1.9 powers per second, and the stimulation intensity was set to 7 times the sensory threshold. We recorded 20 BCR waveforms, calculated the latency period and amplitude of the waveforms, and used the average value for data analysis.

#### 2.2.2. SSR Test

The examination requires a quiet indoor environment with a room temperature of 20°C–25°C. The stimulation electrode was placed on the median nerve of the wrist, the recording electrode was placed on the palm of the hand and the sole of the foot, the reference electrode was placed on the second interosseous muscle of the back of the hand, and the ground electrode was placed on the wrist 15–20 cm. We started the stimulation with a 20 mA stimulation intensity. The stimulation duration was set to 0.1 ms. We stimulated the patients 4 times continuously and measured the average value and amplitude of the initial latency. The unit of the average latency and amplitude is milliseconds (ms) and millivolts (mV), respectively.

### 2.3. Standards for Abnormal Electrophysiological Findings

#### 2.3.1. Criteria for Abnormal Unilateral BCR

The abnormal unilateral BCR findings were as follows: unable to obtain the patient's BCR waveform; the patient's unilateral BCR latency is longer than the sum of the average value of the same-sex control group and 2.5 times the standard deviation (*X* + 2.5 SD); BCR unextracted rate = number of people without BCR/total number of people in the group × 100%; and (4) the patient's unilateral BCR amplitude is smaller than the lowest amplitude of the same-sex control group.

#### 2.3.2. Abnormal SSR Judgment Criteria

The observation indicators are as follows: the latency period of SSR in one limb of the patient is longer than that of the control group on the same side (upper or lower limb; *X* + 2.5 SD) and missing response (waveform is not drawn).

### 2.4. Statistical Analysis

The SPSS 19.0 statistical software was used to analyze and process all data. First, the data are tested for normality and homogeneity of variances; both of which are normally distributed and expressed as mean ± standard deviation (*X* ± SD). A one-way analysis of variance (ANOVA) was used to compare the data among the three groups, whereas the LSD multiple test was used to compare the data between two groups. The chi-square test was used to compare the occurrence and elicitation rates between the two groups. *p* values <0.05 were used to indicate a statistically significant difference.

## 3. Results

### 3.1. Comparison of General Data between the MSA-P, PD, and Control Groups

Age and sex distributions were different among the three groups, but the differences were not statistically significant (*p* > 0.05). There was also no significant difference in the disease course between the PD and MSA-P groups (*p* > 0.05) (Table 1).

### 3.2. The Incidence of Sacral Reflex Dysfunction in the PD and MSA-P Groups

**Figure 1:**
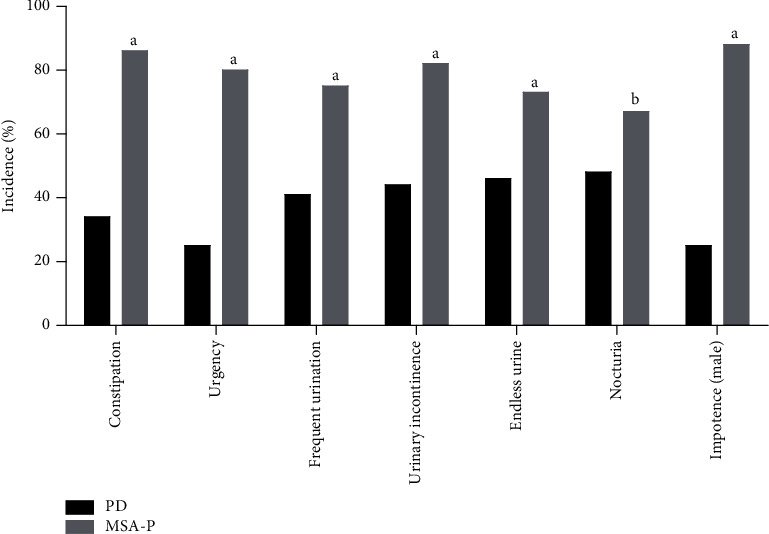
The incidence of sacral reflex dysfunction in the MSA-P group and PD group. ^a^*p* < 0.05 compared to PD. ^b^*p* > 0.05 compared to PD.

The number of PD patients showing clinical manifestations related to sacral reflex dysfunction was 30, which accounts for 51.7% of all PD patients. The most prominent symptom was intractable constipation in 20 patients (34.5%), followed by nocturia, incomplete urination, urgency, urinary incontinence, frequent urination, and male sexual dysfunction (Figure 1).

Altogether, 48 MSA-P patients had clinical manifestations related to sacral nerve damage, which accounts for 92.3% of all MSA-P patients. The most common clinical manifestation was male sexual dysfunction, with 46 cases (88.5%), followed by constipation, urinary incontinence, urinary urgency, frequent urination, incomplete urination, and nocturia.

The proportion of patients with constipation, urgency, frequent urination, urinary incontinence, and male sexual dysfunction were statistically significantly different between the MSA-P and PD groups.

### 3.3. Comparison of the BCR Test Results between the MSA-P, PD, and Control Groups

**Figure 2:**
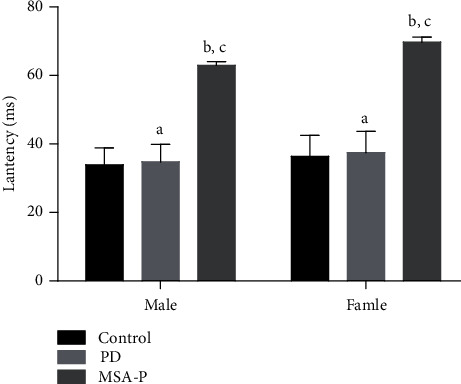
Comparison of BCR latency in the Parkinson's disease group, MSA-P group, and healthy control group. ^a^*p* > 0.05 compared to control. ^b^*p* < 0.05 compared to control. ^c^*p* < 0.05 compared to PD.

**Figure 3:**
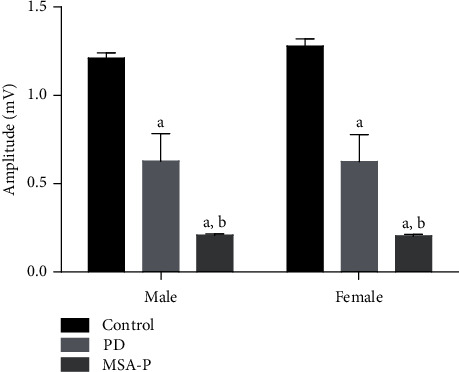
Comparison of BCR amplitude of the PD group, MSA-P group, and control group. ^a^*p* < 0.05 compared to control. ^b^*p* < 0.05 compared to PD.

The BCR elicitation rates of PD and MSA-P patients were 86%and 54%, respectively. The BCR induction rate of the PD group was significantly higher than that of the MSA-P group (Figure 2). The patients from all groups (controls, PD, and MSA-P groups) were paired by sex. The ANOVA test results showed that the difference in the BCR latency period was statistically significant among the three groups. The results of the pairwise comparison using the LSD method showed that the latency period of the MSA-P group was longer than that of the PD (*p* < 0.05) and control (*p* < 0.05) groups, regardless of sex. However, the difference in the latency period between control and PD groups was not statistically significant (Figure 2). The control, PD, and MSA groups were paired by sex and analyzed. The ANOVA test results showed that the difference in BCR amplitude among the three groups was statistically significant. In the pairwise comparison using the LSD method, the results showed that the amplitude of the MSA-P group was lower than that of the PD group (*p* < 0.05), regardless of sex. Moreover, the amplitude of the MSA-P and PD groups was significantly lower than that of the control group (*p* < 0.05) (Figure 3).

### 3.4. Comparison of SSR Average Latency among the MSA-P, PD, and Control Groups

**Figure 4:**
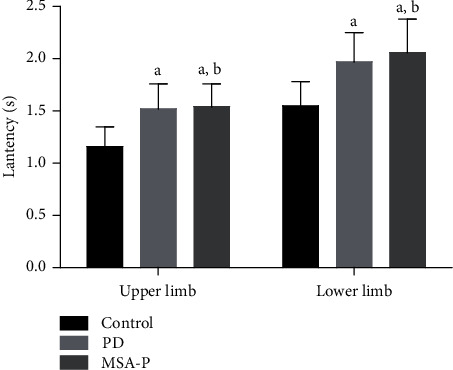
Comparison of SSR average latency between the MSA-P group, PD group, and control group. ^a^*p* < 0.05 compared to control. ^b^*p* > 0.05 compared to PD.

The SSR latency was different among the three groups (*p* < 0.05). The SSR latency of the MSA-P and PD groups was longer than that of the control group. However, there was no significant difference in the SSR latency between the PD and MSA-P groups (*p* > 0.05) (Figure 4).

## 4. Discussion

PD patients often have symptoms of sacral reflex dysfunction, such as sexual dysfunction, bladder dysfunction, and constipation. These symptoms can appear in the preexercise phase of PD, which can seriously affect the daily quality of life of patients [10–12]. It is a well-known fact that most patients with MSA-P have clear and severe sacral reflex dysfunction, with an occurrence rate of 40%–100%, as reported in different studies [13–16]. PD patients can indeed have an autonomic nerve damage similar to that seen in MSA-P patients in clinical settings, but the probability of occurrence is lower in PD patients [17–19]. This finding was also confirmed in our study.

Although there is a difference in the incidence of sacral reflex dysfunction between MSA-P and PD, it is difficult to clinically distinguish individuals from the two diseases based on this incidence. Therefore, establishing an objective evaluation method for sacral reflex dysfunction is very important. SSR is currently a common method for evaluating autonomic nerve function. It has the characteristics of simple operation, low cost, and noninvasive and is not time-consuming. The central nervous part of the SSR reflex arc has not yet been fully elucidated. The incoming nerves of the SSR reflex arc are myelinated fibers deep in the skin, efferent nerves are postsympathetic unmyelinated fibers, and effectors are sweat glands. Therefore, SSR not only reflects the functional status of postganglionic sympathetic nerves but also reflects the functional status of the peripheral afferent fibers and central nerves. Given that there are many factors that can affect the amplitude of SSR and its variability, this study did not detect its amplitude but only used the average value of the SSR latency for the result analysis. In this study, compared with the control group, both the PD and MSA-P groups had the prolonged SSR latency period and missing waveforms, with the difference being statistically significant. However, the difference between the PD and MSA-P groups was not statistically significant, suggesting that SSR cannot be used for the differential diagnosis of PD and MSA-P, which is consistent with the results of Watanabe et al. [20]. This result may be due to the involvement of the sympathetic center in both MSA-P and PD. SSR is a form of reflex arc; thus, a damage to any link in the conduction pathway can cause SSR abnormalities. Therefore, the autonomic nervous function evaluated by using SSR focuses more on integrity, which may be the reason why its differential diagnostic value for both conditions is not significant.

BCR is closely related to rectal, bladder, and sexual functions. After stimulating the pudendal nerve, it is transmitted by the nerve through the spinal cord [21–23], which finally causes the bulbocavernosus muscle to contract. The results of our previous studies showed that MSA-P patients have an abnormal BCR. The present study showed that, compared with the PD and control groups, the MSA-P group had longer BCR latency and decreased amplitude, indicating that the integrity of the BCR arc in patients with MSA-P has been destroyed. The BCR latency of PD patients is not statistically different from that of the control group, whereas the difference in BCR amplitude is statistically different between the two groups. The BCR elicitation rate of the MSA-P group is significantly lesser than that of the PD group, which indicates that the integrity of the BCR arc in PD patients is also impaired, but the spinal cord reflex arc injury in PD patients is less severe than that in MSA-P patients. Sacral spinal cord 2–4 or pudendal nerve damage often occurs in the following two situations [24–26]: presence of local lumbosacral lesions (e.g., cauda equina disease) and dysfunction caused by multisystem neurological degeneration (e.g., MSA-P, PD, cortical basal ganglia degeneration, and DLB). The main pathological basis of the latter is the damage of the Onuf's core [27] of the sacral spinal cord, which has been confirmed in MSA. At present, the mechanism of abnormal BCR in PD patients is still unclear. The possible explanations are as follows: (1) most cases reported in the literature have no pathological confirmation; thus, PD may be misdiagnosed as MSA; and (2) maybe the Onuf core is damaged in these neurodegenerative diseases; however, the severity differs in both groups. Our research data showed that the difference in the degree of BCR abnormality between PD and MSA-P supports the second possibility.

The present study has a major limitation. The number of enrolled MSA-P and PD cases was relatively small. Thus, future studies should accumulate more MSA-P and PD cases.

## 5. Conclusion

Although the MSA-P and PD groups showed prolonged SSR latency and missing waveforms compared to the control group, the difference in the SSR latency and incidence of missing waveforms between the MSA-P and PD groups was not statistically significant; thus, SSR cannot be used for the differential diagnosis of MSA-P and PD. Compared with the PD and control groups, the BCR latency was prolonged and the amplitude was decreased in the MSA-P group, indicating that the integrity of the BCR arc in MSA-P patients was impaired. The BCR amplitude of PD patients is statistically different from that of the control group, suggesting that the integrity of the BCR arc in PD patients is also impaired. The extraction rate of BCR is significantly lower in the MSA-P group than in the PD group, and the degree of damage to the sacral reflex arc is more severe in MSA-P patients than in PD patients.

## Figures and Tables

**Table 1:** Comparison of general data among the MSA-P group, PD group, and control group.

	MSA-P group	PD group	Control group	Statistics	*p* value
Number of cases (*n*)	52	58	30		
Age (years)	52.74 ± 8.32	54.16 ± 7.25	57.16 ± 7.25	*F* = 0.536	0.071
Gender (male %)	28 (54%)	26 (45%)	14 (47%)	*x* ^2^ = 15.735	0.314
Course of disease (months)	29.5	43.7		*t* = 3.52	0.09

## Data Availability

The data used to support the findings of this study are available from the corresponding author upon request.

## Abbreviations

MSA-P: Multiple system atrophy P-type
BCR: Bulbocavernosus reflex
PD: Parkinson's disease
SSR: Sympathetic skin response.
